# People display consistent recency and primacy effects in behavior and neural activity across perceptual and value-based judgments

**DOI:** 10.3758/s13415-025-01285-1

**Published:** 2025-03-26

**Authors:** Minhee Yoo, Giwon Bahg, Brandon Turner, Ian Krajbich

**Affiliations:** 1 Department of Psychology, The Ohio State University, Columbus, OH USA; 2 Department of Psychology, Vanderbilt University, Nashville, TN USA; 3 Department of Economics, The Ohio State University, Columbus, OH USA; 4 Department of Psychology, University of California los Angeles, Los Angeles, CA USA

**Keywords:** Decision-making, Primacy, Recency, Decision neuroscience, Evidence averaging, fMRI

## Abstract

Retrospective judgments require decision-makers to gather information over time and integrate that information into a summary statistic like the average. Many retrospective judgments require putting equal weight on early and late information, in contrast to prospective judgments that involve predicting the future and so rely more on late information. We investigate how people weight information over time when continuously reporting the average stimulus strength in a sequence of displays. We investigate the consistency of these temporal profiles across perceptual and value-based tasks using both behavior and functional magnetic resonance imaging (fMRI) data. We found that people display remarkably consistent temporal weighting functions across choice domains, with a generally strong recency bias and modest primacy bias. The fMRI data revealed evidence-tracking activity in the cuneus in both tasks and in the left dorsolateral prefrontal cortex in the value-based task. Finally, a network of cognitive control regions is more active for people who exhibit a stronger primacy vs. recency bias. Together, our behavioral findings indicate that people consistently overweight recency when evaluating past information, and the neural data suggest that overcoming this tendency may require cognitive control.

**Supplementary Information:**

The online version contains supplementary material available at 10.3758/s13415-025-01285-1.

## Introduction

Many of the decisions that we make require us to gather information over time and integrate that information into a summary evaluation (e.g., the average). For example, we might judge the average quality of a TV show, competence of an employee, or trustworthiness of a politician. Such decisions rely on temporal integration of evidence, from early to recent timepoints.

There are two kinds of judgments that one can make based on past evidence. There are prospective judgments, which involve predicting the future, and retrospective judgments, which involve explaining the past. For example, suppose we are sports analysts tasked with evaluating a team’s performance. We may want to predict whether that team will succeed in the future, e.g., later in the season, in the playoffs, or the following season. Conversely, we may want to determine how well that team has played so far, e.g., to give out awards or positions in the playoffs.

Prospective judgments are well understood; there is a large literature on reinforcement learning and how people should, and do, put more weight on more recent evidence (Rescorla & Wagner, 1972; Sutton & Barto, 1998). In our sports example, playoff outcomes are better predicted by end-of-season performance than early-season performance (Durand et al., 2021; Metz & Jog, 2023).

In comparison, retrospective judgments are less well understood. Do people mistake retrospective judgments for prospective judgments and put too much weight on recent evidence? Or do they jump to conclusions early, putting more weight on early evidence? Anecdotally, retrospective judgments are also overly influenced by recent performance. For example, selections for the college American football playoffs depend heavily on the last games of the season, and selections for the Major League Baseball All Star game, which happens mid-season, depend disproportionately on that season’s performance. Here, we seek a more rigorous investigation of these issues.

Retrospective judgments rely on memory, and memory is known to exhibit primacy and recency effects. When participants are presented with a list of words, they tend to better remember the words from both the beginning (primacy effect) and the end of the list (recency effect) than those from the middle of the list (Murdock Jr., 1962; Oberauer et al., 2018). Primacy and recency effects are not confined to verbal memory. They have been reported across different domains, such as visual and spatial memory (Hurlstone et al., 2014).

In the studies of decision-making and perceptual averaging, there is evidence for primacy and recency effects. In some cases, decision-makers appear to weight the evidence equally over time (Brunton et al., 2013; Wyart et al., 2012), as in Bayesian updating. By contrast, other work has shown that decision-makers put more weight on recent evidence (Cakici & Zaremba, 2023; Cheadle et al., 2014; Do et al., 2022; Ge et al., 2012; Hertwig et al., 2004; Johar et al., 1997; Li & Epley, 2009; Mantonakis et al., 2009; Mohrschladt, 2021; Tong et al., 2019; Tsetsos et al., 2011, 2012; Usher & McClelland, 2001; Wulff et al., 2015, 2018). Conversely, a bias towards early evidence (i.e., primacy) has also been observed (Carney & Banaji, 2012; Hubert-Wallander & Boynton, 2015; Kiani et al., 2008; Mantonakis et al., 2009; Rey et al., 2020; Wilming et al., 2020; Yates et al., 2017; Zylberberg et al., 2012). There are also individual differences; some people choose the option favored early or late in the sequence, whereas others do not show a temporal bias (Pietsch & Vickers, 1997; Tong & Dubé, 2022; Tsetsos et al., 2011; Usher & McClelland, 2001). Although these individual differences have been rigorously studied in the context of perceptual decisions, it is less clear to what degree they exist in value-based decisions.

What also remains unclear is whether these findings on memory and decision-making extend to continuous judgments, such as averaging. With rare exceptions (Do et al., 2022; Hubert-Wallander & Boynton, 2015; Johar et al., 1997; Tong & Dubé, 2022; Tong et al., 2019), primacy and recency biases are inferred from discrete choices. They are typically studied in settings where information must be stored and retrieved later, whereas in continuous judgments the decision-maker need only maintain a running average. It is unknown how the competition between primacy, recency, and optimality combines to form an overall temporal weighting function in estimating average evidence.

It is also unknown whether biases in temporal integration, if any, are consistent across perceptual and value-based domains. There has been growing interest in the commonalities and differences between perceptual and value-based decisions (Frydman & Nave, 2017; Polanía et al., 2014; Shadlen & Shohamy, 2016; Smith & Krajbich, 2021). However, none of this work has looked at continuous judgments.

Finally, the neural mechanisms of evidence averaging are also unknown. There has been influential work studying the accumulation of evidence in discrete choice (Gluth et al., 2012; Hare et al., 2011; Heekeren et al., 2006; Juechems et al., 2017; O’Connell et al., 2018; Pisauro et al., 2017; Rodriguez et al., 2015; Shadlen & Shohamy, 2016). This work has identified input regions—perceptual areas, such as fusiform face area, parahippocampus (Heekeren et al., 2004, 2006) or medial temporal areas (Ho et al., 2009; Kayser et al., 2010), and value-based areas, including vmPFC and striatum (Gluth et al., 2012; Hare et al., 2011; Pisauro et al., 2017; Rodriguez et al., 2015), as well as accumulator regions including (pre-) motor cortex, dlPFC, and parts of parietal cortex (Liu & Pleskac, 2011; Turner et al., 2019; Wilming et al., 2020). The input regions reflect the nature of the stimuli being evaluated, whereas the accumulator regions largely reflect the response modality. Despite this influential work, there has not been any investigation of how evidence is averaged over time in the brain.

We study the ways in which people temporally weight evidence when forming perceptual and value-based judgments. We investigate individual differences in temporal weighting functions and how these individual differences arise in the brain. We use a modified interrogation paradigm (Bahg et al., 2020) in which subjects are asked to continuously report their estimate of the average evidence as new evidence becomes available, analogous to how college football rankings are updated each week after the latest set of games. In the original interrogation paradigm (Ratcliff, 2006; Turner et al., 2017; Usher & McClelland, 2001), a response cue is presented after a delay following the presentation of a stimulus. Subjects are instructed to make a single choice immediately after the response cue. In contrast, in the modified interrogation paradigm, subjects continuously report their estimates of average evidence throughout a trial. These ongoing measures allow us to investigate how temporal biases impact the estimates of average evidence. Moreover, this paradigm allows us to identify brain regions that track the average evidence by correlating the reports with fMRI signals.

To preview the results, we find that people show considerable recency bias and some primacy bias. These temporal weighting functions are highly consistent within an individual, showing strong correlations between perceptual and value-based tasks. As expected, parietal cortex and the fronto-striatal reward network encode the inputs for perceptual and value-based tasks, respectively, while only the dorsolateral prefrontal cortex (dlPFC) represents the averaged evidence, and only in the value-based task. Finally, we find that people who exhibit more primacy bias compared to recency bias display more activity in the cognitive control network including the intraparietal sulcus and dlPFC.

## Methods

### Subjects

Forty-six subjects participated in this study (23 males, mean age 21.4 years). Subjects were recruited from The Ohio State University Experimental Economics Pool. They received $35 as a base payoff and could earn an additional payoff up to $10. All subjects were right-handed and had normal or corrected-to-normal vision.

We excluded eight subjects from the analyses. One subject had a structural abnormality in their brain, and six subjects moved their head more than 3 mm. We additionally excluded one subject owing to failure to understand the task, based on a postexperiment questionnaire where they indicated that they tracked the difference between single pairs of stimuli rather than the average.

## Experimental procedure

### Rating task

Subjects completed two tasks. In the first task, which took place outside the MRI scanner, they rated 144 snack foods on a continuous scale from 0 (least liked) to 10 (most liked). Before rating the foods, subjects saw all of them in a slideshow. After the slideshow, subjects saw one food at a time and rated how much they would like to eat it at the end of the experiment. They used the mouse to move a slider along a rating scale and clicked the left mouse button to mark their ratings.

### Evidence-averaging task

After the rating task, subjects completed an evidence-averaging task in the MRI scanner, which took approximately 50 min to complete. The evidence-averaging task consisted of two blocks of a perceptual task and two blocks of a value-based task. The two tasks alternated. The order of the blocks was counterbalanced across subjects. Each block had 15 trials.

**Fig. 1:**
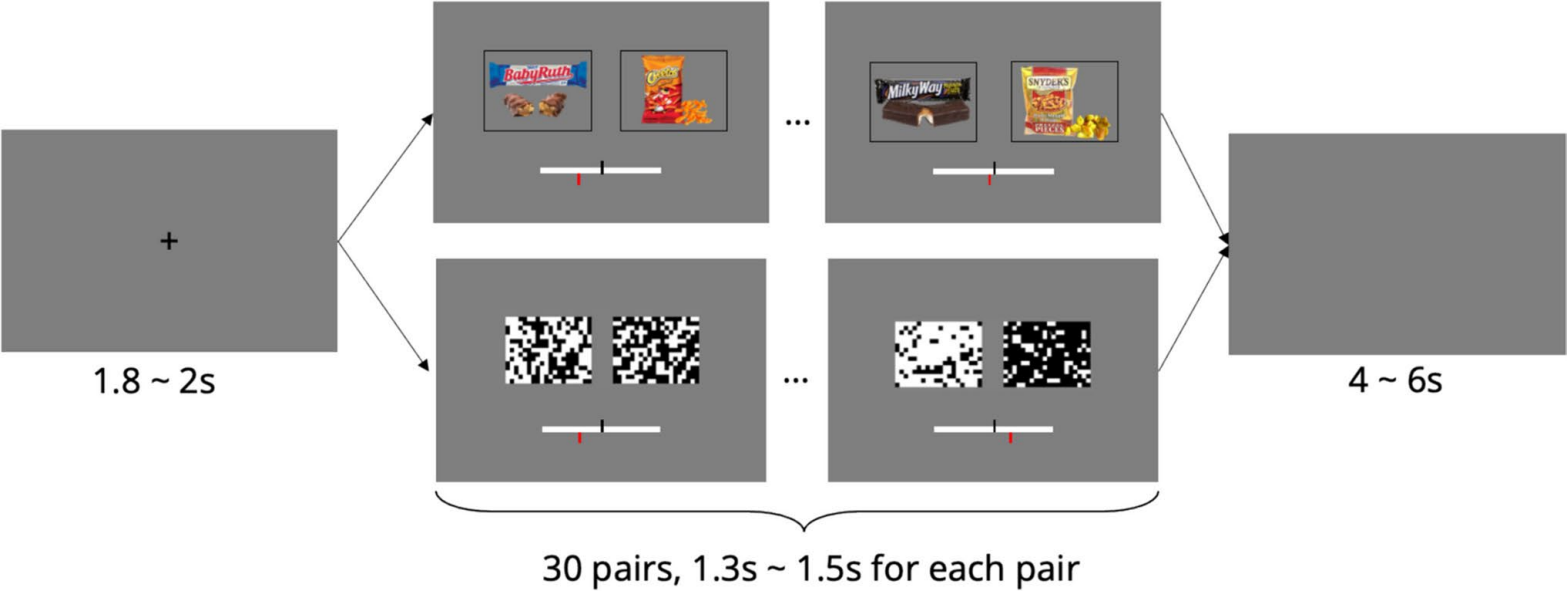
Timeline of the evidence-averaging task. After a fixation cross, subjects saw 30 pairs of stimuli in a trial. They saw each pair for 1.3 to 1.5 s. Square grids and snack foods were presented in the perceptual and value-based tasks, respectively. Subjects continuously reported the average difference between the right and left sides by moving the red slider bar along the scale with a joystick. The center of the scale was marked with a black vertical bar. At the end of each trial, there was a 4- to 6-s delay before the next trial

In each trial, subjects saw 30 pairs of stimuli in series (Fig. 1). Each pair was presented for 1.3 to 1.5 s. The stimuli were grids of black and white squares in the perceptual task and snack foods in the value-based task. While watching each series of stimuli, subjects continuously reported the average difference between the right and left stimulus by using a joystick (Current Designs Tethyx) to move a slider bar along a scale. Specifically, they reported which side of the screen had on average more white squares or better foods, and by how much.

There were no response cues during the task. The slider began each trial at the center of the scale. Subjects moved the slider whenever they thought the average difference had changed. The slider remained in the same position until subjects moved the joystick. The tilt of the joystick determined how fast the slider moved in the tilted direction. We recorded the slider position at 30 Hz.

Before the MRI task, subjects learned how to map stimulus differences to positions on the scale. Subjects first learned the meaning of the endpoints on the scale. In the perceptual task, they were informed that the endpoints indicated that one side had 60% more white dots. In the value-based task, they were informed that the endpoints indicated that foods on that side were better by x (the rounded maximum rating difference between two foods) out of 10. Once subjects learned the meaning of the scales’ endpoints, we displayed several examples where we moved the slider from the left end to the right end of the scale and showed corresponding stimulus pairs. We showed three examples at nine points on the scale for 1.5 s each. After seeing the examples, subjects practiced a trial without feedback.

Subjects earned money based on their performance in the evidence-averaging task. We determined their earnings based on the deviation of the slider from the correct answer at the end of each stimulus pair, averaged across all stimulus pairs. The correct answer was the average of all the stimuli presented up to that time in a trial. Subjects earned the maximum and minimum bonus when the slider deviated from the correct answer by less than 4% and 20% of the scale length, respectively. The bonus decreased gradually from $10 to $1 between these extremes. Subjects earned $2.63 on average (standard deviation = 2.28).

### Sequence

**Fig. 2:**
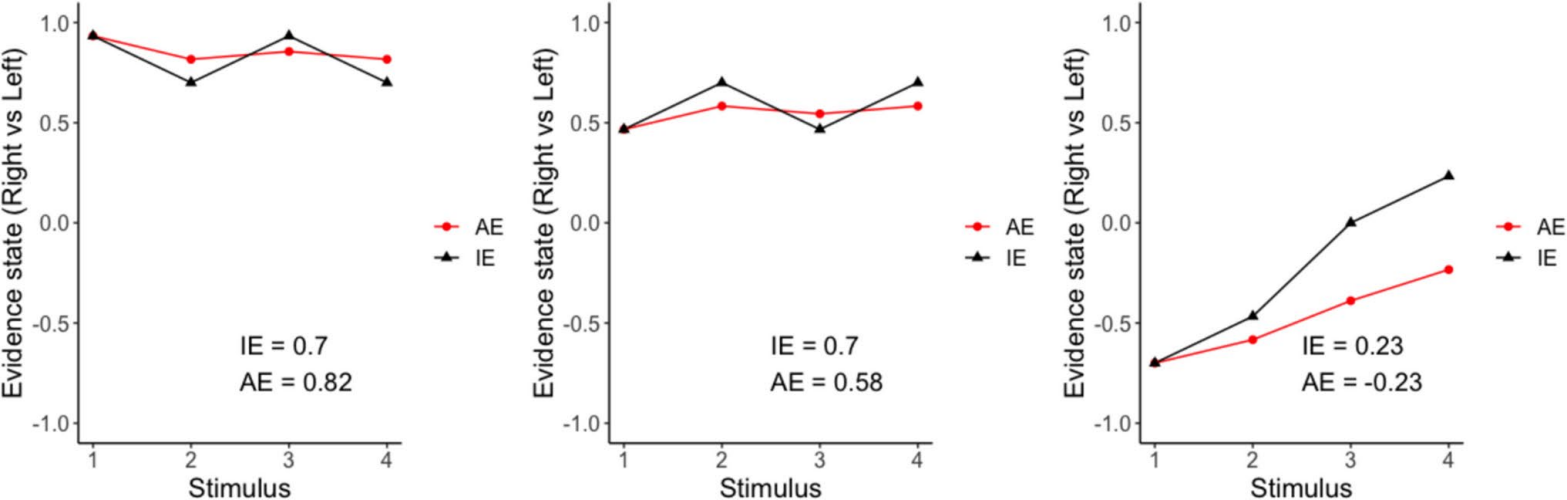
The difference between instantaneous evidence (IE) and average evidence (AE). Time course examples of the first four pairs of stimuli in three trials. Each panel shows how IE and AE change over time in a trial. IE (black) represents the difference between a pair of stimuli at the current point in time. AE (red) represents the average evidence up to that point in time. In other words, the red AE curve is the cumulative mean of the black IE curve. The IE and AE written in each panel represent their values after the fourth stimulus pair

We generated two block-length sequences to present stimuli in each task. Each sequence was designed to minimize the correlation between the instantaneous evidence (IE) and average evidence (AE), i.e., the mean of IE. The IE is the difference in the number of white squares (perceptual) or subjective values (value-based) between the two stimuli on the screen, right minus left. The IE had nine levels, in arbitrary units from − 0.4 to 0.4, increasing by 0.1. To generate a trial-level sequence, we simulated a random walk process with 30 steps corresponding to 30 stimulus pairs in a trial. We used a random walk process to generate stimulus sequences to ensure that the input would gradually change over time. This gradual change allowed the AE to evolve smoothly, which made it easier for subjects to update their AE and report it using a joystick. The initial value was drawn from the nine possible IE levels. We determined the direction of the random walk by sampling a value from $Uniform\left(\text{0,1}\right)$ and calculating whether it was greater or smaller than 0.5. Given the direction, we determined the step size by sampling a value from [$Poisson\left(0.3\right)+1]/10$. Examples of the sequences for the first four stimulus pairs in a trial are illustrated in Fig. 2.

We created 15 trial-level sequences per block. We generated 100 block-level sequences by repeating this process and then selected the one with the lowest correlation between IE and AE. We generated two such sequences and used them in both perceptual and value-based tasks, in the same order. The order of the block-level sequences was fixed within each subject but was counterbalanced across subjects.

### Stimulus

In the perceptual task, we manipulated the difference in the number of white squares between the two grids. Each grid had 300 squares, with 15 rows and 20 columns. The size of each square was 20 × 20 pixels. The total number of white squares on the screen was constant; when we increased the number of white squares in one grid, we also decreased it by the same amount in the other grid. When IE was 0, half of each grid was white and the other half was black. As the magnitude of IE increased, we increased the percentage of white in one grid by 7% and decreased the percentage of white in the other grid by 7%. This resulted in difference levels of 0%, 14%, 28%, 42%, and 56% of the total number of white squares between the two grids. The endpoint of the slider scale roughly corresponded to the maximum difference between the two grids, namely 60%.

In the value-based task, we manipulated the rating difference between the two foods. We chose stimuli based on each subject’s ratings. We excluded the lowest and highest rated items (5% each), including all items rated 0 or 10. We then computed the rating differences between all pairs of the remaining snack foods. Next, we evenly divided the range of value differences into nine bins, which were then matched with the nine levels of IE. Stimulus pairs from each bin were drawn according to the specified sequence. The endpoint of the scale was customized for each subject and corresponded to the rounded maximum rating difference between two foods.

For behavioral data analysis, we rescaled the position of the slider, the rating difference between snack foods, and the difference in the number of white squares between grids to be in the range of − 1 to + 1. This rescaling process made it easier to compare results between the two decision domains and fit computational models to the data.

## Behavioral data analysis

### Accuracy

We first assessed how well subjects tracked the AE. If subjects were able to perfectly average all evidence without any noise in the evidence-averaging process, their responses would align with the running average of the IE (simple AE). To quantify the accuracy of subjects in the evidence-averaging task, we computed the mean error (ME). Specifically, ME was calculated as the mean absolute difference between the last slider position ${y}_{b,l,s}$ of stimulus pair *s* in trial *l* of block *b* and the simple ${AE}_{b,l,s}$: $\frac{1}{N}{\sum }_{b}{\sum }_{l}{\sum }_{s}\left|{y}_{b,l,s}-{simple AE}_{b,l,s}\right|$ where ${simple AE}_{b,l,s}=\sum_{s}I{E}_{b,l,s}/{N}_{s}$. Here, *N* refers to the total number of stimulus pairs (i.e., 2 blocks per decision domain × 15 trials per block × 30 pairs per trial = 900 pairs), and ${N}_{s}$ refers to the number of stimulus pairs presented up to that time in a trial. We computed ME for each subject on each task.

Next, we examined how well subjects tracked AE changed over time. If subjects showed a temporal bias, their responses would deviate more from the simple AE as time passed within a trial. To test this possibility, we ran a linear regression on the error in the evidence-averaging task: $\left|{y}_{b,l,s}-{simple AE}_{b,l,s}\right|$. Time within a trial, task type (value-based vs. perceptual), and their interaction were included as regressors. Standard errors were clustered at the subject level.

Lastly, we examined how sensitive subjects were to IE and whether the sensitivity to IE changed over time. When computing the average, the influence of IE on AE decreases over time. Thus, we expect to see a decreasing effect of IE on AE as time passes within a trial. To test this possibility, we ran a linear regression on AE updates in response to each IE within a trial. AE updates were defined as the difference between the last slider positions of two consecutive stimulus pairs: ${y}_{b,l,s}-{y}_{b,l,s-1}$. IE ($I{E}_{b,l,s}$), time within a trial, task type (value-based vs. perceptual), and all their interactions were included as regressors. Standard errors were clustered at the subject level.

### Deviance index

Next, we examined temporal bias in the evidence-averaging process. Specifically, we assessed the degree to which subjects exhibited recency bias. At one extreme a subject could perfectly report the AE, while at the other extreme a subject could discard past information and simply report the IE (Fig. 2). IE and AE are the same at the beginning of the trial but diverge over time. To quantify how closely subjects' responses aligned with AE vs. IE, we computed the ratio of how much the last slider position ${y}_{b,l,s}$ of stimulus pair *s* in trial *l* of block *b* deviated from IE vs. AE:

$$d_a=\frac{\sum_b\sum_l\sum_s\left|y_{b,l,s}-{IE}_{b,l,s}\right|}{\sum_b\sum_l\sum_s\left|y_{b,l,s}-{simpleAE}_{b,l,s}\right|}.$$

We first computed the deviation of the last slider position from IE and AE for each stimulus pair, then summed up those deviations, and then computed the ratio between the two sums. This deviance index (${d}_{a}$) quantifies the overall response deviation from IE relative to AE. The deviance index has a range from 0 to infinity, with larger values corresponding to better performance, i.e., better tracking of AE. We computed *d*_*a*_ for each subject on each task.

### Averaging Diffusion Model

We used the Averaging Diffusion Model (Turner et al., 2017) to investigate how subjects computed AE over time. The ADM is a modification of the Drift Diffusion Model (DDM). The ADM assumes that a decision maker integrates noisy samples of evidence to estimate the mean of that evidence. The ADM explains how the estimate of the average evidence evolves over time and links it to a decision maker’s response at any given time.

To calculate the decision variable of the ADM at time *t* (denoted $\mu \left(t\right)$), we used a weighted average of IE. Specifically, when a total of *s*_*t*_ stimulus pairs have been presented up to time *t* and the weight on IE of the *i*-th stimulus pair is ${w}_{{s}_{t}, i},$ the ADM’s decision variable is a weighted average of IE presented up to that time: $\mu \left(t\right)={\Sigma }_{i=1}^{{s}_{t}}{w}_{{s}_{t},i}I{E}_{i}/{\Sigma }_{i=1}^{{s}_{t}}{w}_{{s}_{t},i}$. Note that *i* indexes stimulus pairs while *t* indexes measurements of the slider position. The weight on the *i-*th stimulus pair is determined by a temporal weighting function (Galdo et al., 2022; Pooley et al., 2011): ${w}_{{s}_{t},i}=\left[1-\left(1-{\varepsilon }_{primacy}^{i}\right)\left(1- {\varepsilon }_{recency}^{{s}_{t}-i+1}\right)\right]\left(1-\eta \right)+\eta .$ Since *s*_*t*_ is updated with each new stimulus pair, the weight on each stimulus pair and the ADM’s decision variable are updated every time a new IE is presented. A primacy parameter ${\varepsilon }_{primacy}$ and a recency parameter ${\varepsilon }_{recency}$ determine the weights on early and recent IE. The lowest possible weight on a stimulus pair is determined by *η*. We set *η* as 0.01 and estimated ${\varepsilon }_{primacy}$ and ${\varepsilon }_{recency}$. The temporal weighting function determines the weights on early and recent decision evidence relative to the baseline *η*. As long as the baseline is low, ${\varepsilon }_{primacy}$ and ${\varepsilon }_{recency}$ can adjust to match an individual’s temporal weighting function. Thus, we fixed *η* and estimated only ${\varepsilon }_{primacy}$ and ${\varepsilon }_{recency}$.

The ADM can represent two mechanisms for updating the AE. First, it can represent a process in which subjects recompute the AE every time a new IE is presented. When a new IE is presented, the weights on each IE are updated using a temporal weighting function and the AE is recomputed with the updated weights and the IEs stored in memory. Second, under a certain constraint, the ADM can represent a process in which subjects maintain their current estimate of AE and update it with each new IE. The AE in the ADM at time *t* can be re-expressed as:$AE\left(t\right)=\frac{{\Sigma }_{i=1}^{{s}_{t}-1}{w}_{{s}_{t},i}I{E}_{i} + {w}_{{s}_{t},{s}_{t}}I{E}_{{s}_{t}}}{{\Sigma }_{i=1}^{{s}_{t}}{w}_{{s}_{t},i}}$. When the weight on each IE is fixed and does not change over time (${w}_{1,i}={w}_{2,i}\dots {w}_{30,i}={w}_{i})$, the AE in the ADM can be reformulated as:$AE\left(t\right)=\frac{{\Sigma }_{i=1}^{{s}_{t}-1}{w}_{i}}{{\Sigma }_{i=1}^{{s}_{t}}{w}_{i}}A{E}_{{s}_{t}-1}+\frac{{w}_{{s}_{t}}}{{\Sigma }_{i=1}^{{s}_{t}}{w}_{i}}I{E}_{{s}_{t}}$. This formulation represents the process of maintaining the most recent estimate of AE and updating it with each new IE.

The measured average evidence at time *t* (i.e., the position of the slider), $E\left(t\right),$ follows a normal distribution $E\left(t\right) \sim Normal\left(\mu \left(t\right), {\sigma }^{2}\left(t\right)\right)$. $\sigma \left(t\right)$ represents the standard deviation of the average evidence. As the subject collects more evidence, their estimate of the AE becomes increasingly precise. Thus, the standard deviation of AE decreases over time: $\sigma \left(t\right)={\sigma }_{w}/\sqrt{t}$. Here, ${\sigma }_{w}$ is the standard deviation of within-trial variability in the samples of evidence.

We compared the ADM fits to subjects’ AE reports in the tasks. To do so, we computed the likelihood of the slider position ${y}_{b,l,t}$ at time *t* in trial *l* of block *b*, given the distribution of the measured average evidence at that time:

$$L\left(\theta |D\right)= \prod_{b}\prod_{l}\prod _{t}Normal\left({y}_{b,l,t}|{\mu }_{b,l}\left(t\right), {\sigma }_{b,l}^{2}\left(t\right)\right)$$

where θ is a generic notation for the ADM parameters. We evaluated this likelihood for all recorded slider positions, except for those in the first stimulus pair of each trial. These initial measurements were excluded to account for the large movements that are initially required in the task, coupled with the sluggish nature of moving the slider with the joystick.

We constructed four variants of the ADM based on hypotheses about temporal bias and noise in the evidence-averaging process. First, we tested whether the temporal bias is consistent across the two tasks or not. To test this hypothesis, we constructed two models: a separate-temporal-bias model and a common-temporal-bias model. In the separate-temporal-bias model, we separately estimated ${\varepsilon }_{primacy}$ and ${\varepsilon }_{recency}$ for the perceptual task and the value-based task. In contrast, we estimated a single ${\varepsilon }_{primacy}$ and ${\varepsilon }_{recency}$ across the two tasks in the common-temporal-bias model.

Next, we considered whether the noise in the evidence-averaging process differs between the perceptual task and the value-based task. We constructed two models, a separate-noise model and a common-noise model, to test this hypothesis. In the separate-noise model, ${\sigma }_{w}$ was allowed to differ between the perceptual task and the value-based task. However, the common-noise model assumes a single noise parameter for both tasks, and thus only one ${\sigma }_{w}$ was estimated across the two tasks.

We fitted four models to each subject’s data by considering all possible combinations of these hypotheses (Table S1). To determine the better model for each subject, we compared the performance of the four models using the widely applicable information criterion (WAIC; Vehtari et al., 2017; Watanabe, 2010). The model with the lower WAIC was chosen as the preferred model for that subject.

We fitted these models to each subject’s data using STAN (Stan Development Team n.d.). We set $Beta\left(\text{2,4}\right)$ as the prior of ${\varepsilon }_{primacy}$ and ${\varepsilon }_{recency}$, and $Normal\left(-1, 1\right)$ as the prior of $\text{log}({\sigma }_{w})$. In the first fitting attempt, we collected 4000 samples from each of the four chains after discarding the first 1000 samples. Then, we computed  $\widehat{R}$ values (Bürkner et al., 2023; Vehtari et al., 2021) to assess the convergence of the chains. $\widehat{R}$ values serve as indicators of convergence in posterior chains. Ideally, $\widehat{R}$ values should be close to 1, indicating that the chains have converged. $\widehat{R}$ values were higher than 1.01 for some subjects, suggesting a divergence of chains. Divergence was observed in 14 subjects in the separate-temporal-bias and separate-noise model, 9 subjects in the common-temporal-bias and separate-noise model, 12 subjects in the separate-temporal-bias and common-noise model, and 10 subjects in the common-temporal-bias and common-noise model. To address this issue, we re-fitted the models for subjects where divergence occurred. We initialized chains with informed initial values in this case. We selected the chain that had the highest log posterior probability. Then, we drew random values from the 95% highest-density interval (HDI) of parameters of the selected chain to initialize chains in the second fitting. In the second fitting, we used four chains and collected 4000 samples from each chain after discarding 1000 samples. After the second fitting, $\widehat{R}$ values of all parameters were smaller than 1.01, indicating a successful convergence.

## MRI data acquisition and analysis

We collected the functional and structural MRI data at the Center for Cognitive and Brain Imaging at The Ohio State University. We used a 32-channel head coil and 3 T MRI scanner (MAGNETOM Tim Trio; Siemens Medical Solutions). We first collected structural MRI data [repetition time (TR) = 2400 ms; echo time (TE) = 2.22 ms; flip angle = 8 degree; 208 slices; voxel size 0.8 × 0.8 × 0.8 mm; 300 × 320 matrix size]. Then, we collected four runs of functional MRI data (740 volumes per run, around 12 min). Functional MRI data was acquired with a multiband echo-planar imaging sequence [TR = 1000 ms; TE = 28 ms; flip angle = 60 degree; 45 interleaved slices, voxel size 3 × 3 x 3 mm, 72 × 72 matrix size; multiband acceleration factor = 3]. We tilted the acquisition plane 15 degrees upwards from the line connecting the anterior commissure and posterior commissure to reduce signal dropout in the orbitofrontal cortex (Deichmann et al., 2003). A field map was collected to correct the spatial distortion caused by inhomogeneity of the magnetic field [TR = 670 ms; TE1 = 5.19 ms; TE2 = 7.65 ms; flip angle = 60 degree; 60 slices; voxel size 3 × 3 × 3 mm; 72 × 72 matrix size].

Stimuli were presented using MATLAB Psychtoolbox (Brainard, 1997; Kleiner et al., 2007; Pelli, 1997) in MATLAB and displayed with a DLP projector onto a screen.

### Preprocessing

We preprocessed and analyzed the MRI data with Statistical Parametric Mapping 12 (SPM12; the Wellcome Trust Centre for Neuroimaging, University College London, UK). We corrected the different slice acquisition time of the functional MRI data. Then, we realigned and unwarped functional MRI data to correct head movements and spatial distribution using a voxel displacement map. The voxel displacement map was created by processing the field map with the field map toolbox. The structural MRI data was normalized to the MNI space by a unified segmentation. Structural MRI data was coregistered to the functional MRI data. Functional MRI data was normalized to the MNI space by using the deformation field used to normalize structural MRI data into the MNI space. We smoothed the normalized functional MRI data with an 8-mm full width at half maximum Gaussian kernel.

### Statistical analysis

We constructed two generalized linear models (GLMs) to identify brain regions tracking AE and IE. Each block was modeled with four regressors and six realignment parameters that were generated during preprocessing.

### GLM 1

The first regressor was the onset of each stimulus pair with a duration of zero. This regressor had three parametric modulators: unsigned IE, unsigned AE, and the joystick position. Unsigned IE is the absolute difference between stimuli in a pair (normalized to lie between 0 and 1). Unsigned AE is the absolute AE predicted by the ADM with separate temporal weighting functions and common noise for both tasks. The joystick position captures how much the joystick was tilted from the center (normalized to lie between 0 and 1). We used the first sampled joystick position after stimulus presentation. We turned off orthogonalization of parametric modulators. All regressors were modeled with a canonical hemodynamic response function without temporal or dispersion derivatives.

### GLM 2

Regressors in GLM2 were similar to those in GLM1, except that instead of unsigned AE, it included subjects’ responses, specifically their absolute final slider position for each stimulus pair. If the slider was at the center or at either end of the scale, this variable was zero or one, respectively. Note that the tilt of the joystick determined how fast the slider moved in the tilted direction and not the position of the slider. Thus, subjects’ responses (slider position) and the joystick position were not usually the same.

### Both GLMs

We created four contrast images for each GLM. For each task, we made a positive contrast image for the unsigned IE regressor and a positive contrast image for the unsigned AE regressor. We ran a one-sample *t*-test with each contrast image at the group-level. To correct for multiple comparisons, we applied a threshold of corrected *p* < 0.05. We defined clusters as *p* = 0.001 and further corrected the *p*-values based on the size of clusters (Woo et al., 2014) or the peak activation of a cluster.

### ROI analysis

We explored the neural mechanisms of temporal bias using a ROI analysis. In our task, the influence of each new sample on the average decreases as a trial progresses. As a result, a decision maker may adopt a strategy that prioritizes early samples and downweights new samples. This strategy leads to a primacy bias. Thus, primacy bias may arise from the activity of brain regions involved in inhibiting the updates of the average, such as brain regions related to cognitive control.

We defined ROIs using a term-based meta-analysis at Neurosynth (Yarkoni et al., 2011). We used the association test to create a brain map that is preferentially related to “cognitive control.” Based on the meta-analysis map, clusters in bilateral intraparietal cortex (Left: x =  − 32, y =  − 56, z = 44; Right: x = 52, y =  − 45, z = 46) and bilateral dorsolateral prefrontal cortex (Left: x =  − 44, y = 20, z = 30; Right: x = 44, y = 17, z = 34) were selected as our ROIs. Then, we extracted the beta estimates of the stimulus onset regressor in the GLM1 using the MarsBaR package (Brett et al., 2002) in SPM. Beta estimates of the stimulus onset regressor were correlated with the mean of the difference between primacy and recency parameters for each decision domain. We also examined the correlation between beta estimates of the stimulus onset regressor the mean of primacy and recency parameter, respectively (Table S7).

## Results

### Accuracy

We used the ME to evaluate subjects’ performance in the evidence-averaging task. If a subject had no temporal bias and successfully averaged all the evidence without any noise in the evidence-averaging process, their responses would follow the simple AE, resulting in an ME of zero. Therefore, lower ME values indicate better tracking of the simple AE due to a smaller temporal bias and less noise in the evidence-averaging process.

In both the perceptual and value-based tasks, the ME was larger than zero (one sample *t*-test, Perceptual task: t(37) = 19.19, *p* = 10^–21^; Value-based task: t(37) = 20.88, *p* = 10^–22^). The average ME across subjects was 0.27 for both tasks. This means that, on average, subjects deviated from the simple AE by approximately 13% of the length of the scale. Notably, there was no significant difference in performance between the two decision domains (paired *t*-test, t(37) = 0.58, *p* = 0.57).

The deviation of subjects’ responses from the simple AE grew over time in a trial (Table S8). Time had a positive effect on the error in the evidence-averaging task (time: β = 0.003, *p* = 0.0004). The error was initially larger in the value-based task (task type value-based: β = 0.02, *p* = 0.001) but increased more slowly than in the perceptual task (interaction of time and task type value-based: β =  − 0.002, *p* = 0.0001).

Subjects became less sensitive to IE over time within a trial when computing AE (Table S9). IE had a positive effect on AE updates (IE: β = 0.18, *p* < 10^–16^), and the positive effect of IE on AE decreased over time within a trial (interaction of IE and time: β =  − 0.005, *p* < 10^–16^). The effect of IE on AE was smaller in the value-based task (interaction of IE and task type value-based: β =  − 0.04, *p* = 10^–11^), but decreased less over time than in the perceptual task (interaction of IE, time, and task type value-based: β = 0.001, *p* = 10^–11^).

## Deviance index

We used a deviance index to quantify temporal bias in the evidence-averaging task. The deviance index ranges from 0 to infinity. The higher deviance index means that subjects were tracking the simple AE rather than tracking the IE. A deviance index of 1 corresponds to being equally distant from the simple AE and the IE, whereas a deviance index < 1 corresponds to being closer to the IE than the simple AE. Thus, a deviance index < 1 indicates behavior more in line with reporting the evidence from only the most recent sample.

**Fig. 3:**
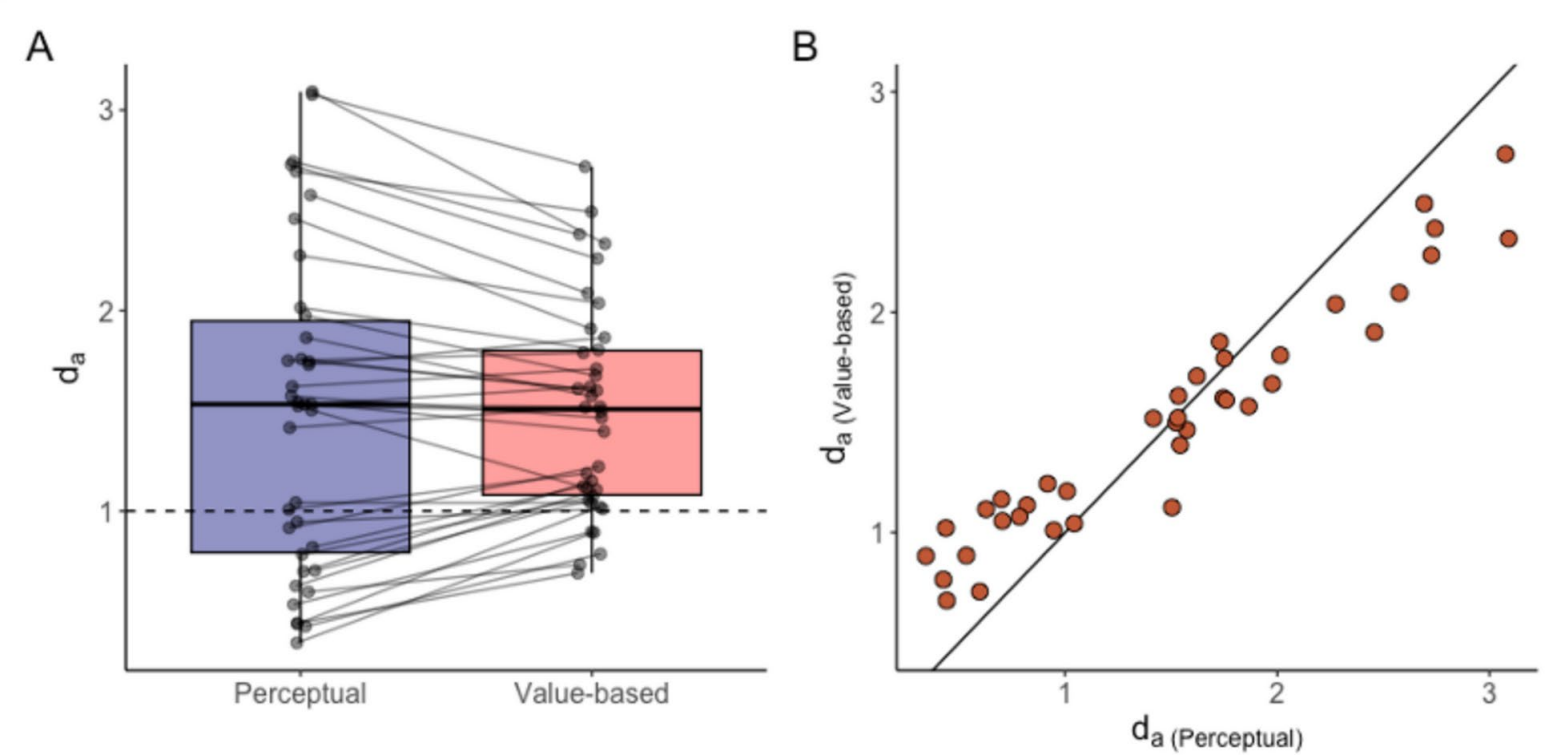
Deviance index. (**A**) Deviance index for the perceptual and the value-based tasks. Each dot represents the deviance index of an individual subject, and each line connects an individual subject’s deviance index across the two tasks. (**B**) Scatter plot of the deviance index between the two tasks. Each dot represents the deviance index of an individual subject. The line represents the identity line (y = x)

Individual differences in temporal bias were evident in our study. The deviance index was significantly higher than 1 in both tasks (M_Perceptual_ = 1.50, M_Value-based_ = 1.50; one-sample *t*-test, Perceptual task: t(37) = 3.87, *p* = 10^–4^; Value-based task: t(37) = 5.91, *p* = 10^–7^; Fig. 3A). This indicates that, on average, subjects tended to track the AE more than the IE.

However, the range of the deviance index was considerable, varying from 0.34 to 3.09 in the perceptual task and from 0.69 to 2.72 in the value-based task. Notably, 13 subjects in the perceptual task and five subjects in the value-based task had deviance indexes less than one, showing a strong recency bias.

Despite significant individual differences in temporal bias, there was remarkable consistency within an individual across tasks. The deviance index in the perceptual task was not significantly different from the deviance index in the value-based task (paired *t*-test, t(37) = 0.04, *p* = 0.97; Fig. 3A). Additionally, there was a very strong positive correlation between the deviance indexes for the two tasks (r(36) = 0.96, *p* < 10^–16^; Fig. 3B). This shows that subjects with a strong recency bias in the perceptual task also had a strong recency bias in the value-based task.

## Averaging Diffusion Model

**Fig. 4:**
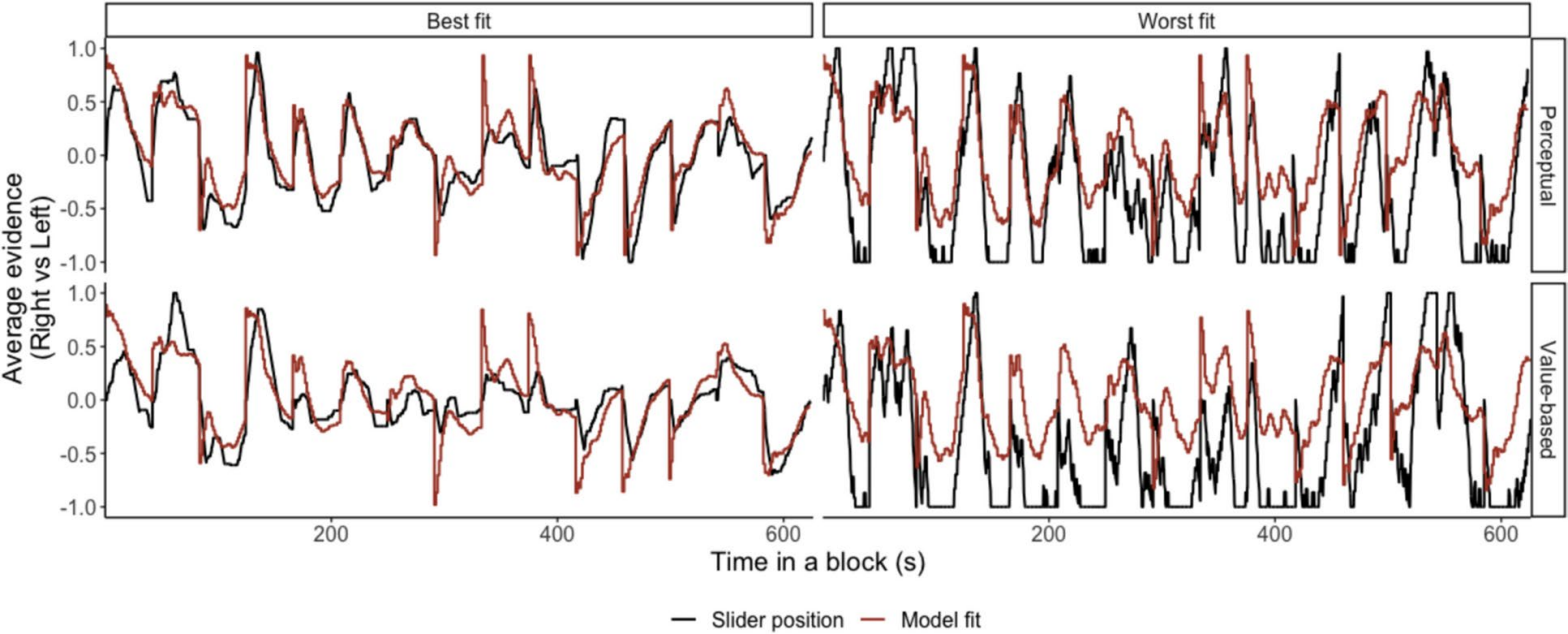
Model fits. The figure shows the responses of two subjects, i.e., slider positions on the scale (black line), and fits of the separate-temporal-bias model (red line). The two subjects’ data from a block of perceptual and value-based tasks are displayed. The two blocks share the same stimulus sequence. These subjects had the lowest (left column, best fit) and highest (right column, worst fit) WAIC to illustrate how well the Averaging Diffusion Model (ADM) explains the data. The mean of posterior distributions was used to generate the model fits of the ADM

Among the four models considered, the model assuming separate temporal bias and separate noise for perceptual task and value-based task provided the best fit for all subjects based on the WAIC goodness-of-fit metric (see Fig. 4 for model fits and Table S1 for WAIC). This indicates that the exact shape of the temporal weighting function differed between the two tasks, and the noise in the evidence-averaging process also varied across tasks.

To further investigate temporal bias using the ADM, we examined the posterior distributions of parameters from the separate-temporal-bias and separate-noise model (Table S2). We quantified temporal bias by computing the difference between ${\varepsilon }_{primacy}$ and ${\varepsilon }_{recency}$ in each task (i.e., ${\varepsilon }_{primacy}-{\varepsilon }_{recency}$). Then, we assessed whether the 95% HDI of ${\varepsilon }_{primacy}-{\varepsilon }_{recency}$ was strictly positive or negative, and whether ${\varepsilon }_{primacy}-{\varepsilon }_{recency}$ was correlated between tasks.

**Fig. 5:**
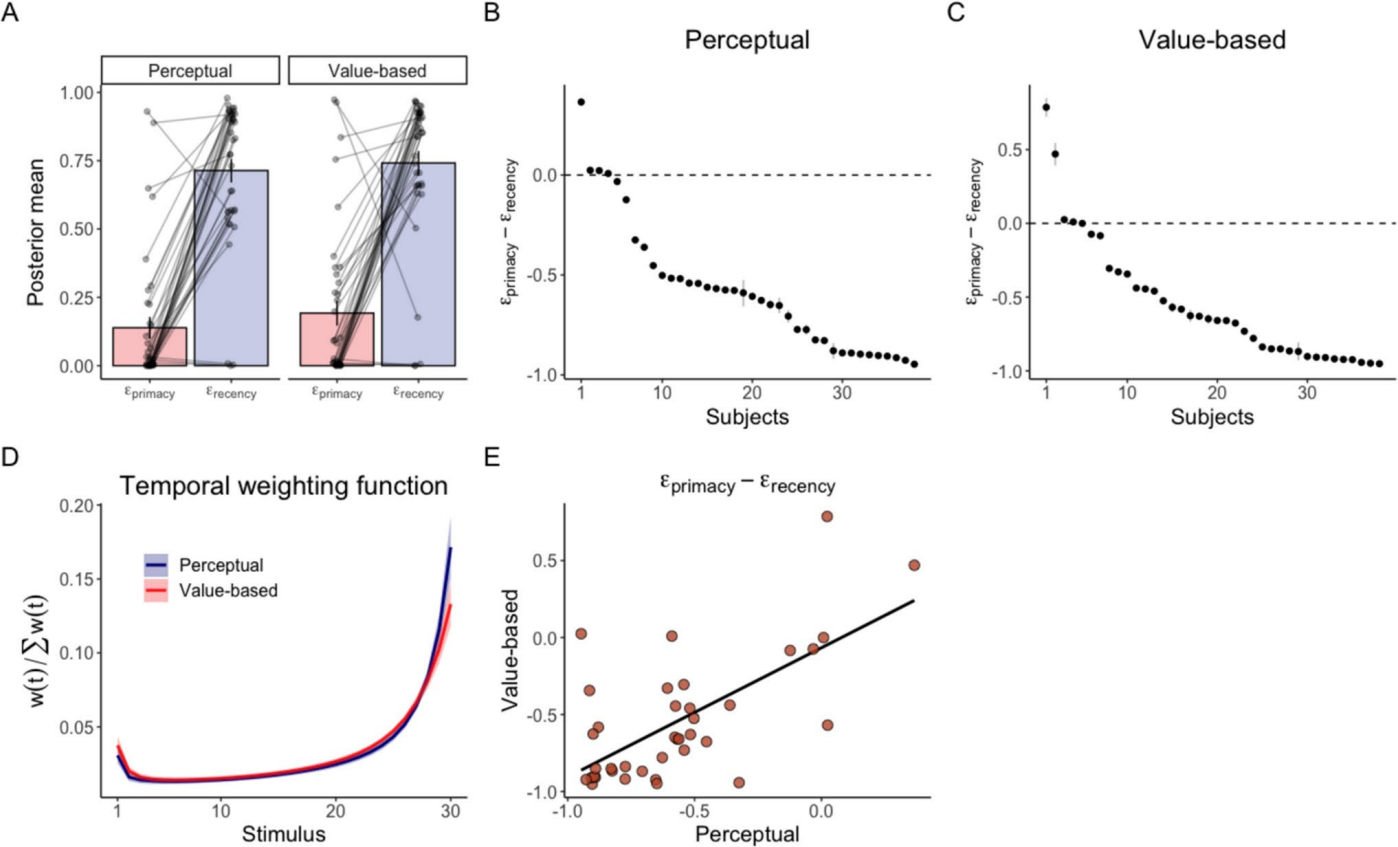
**Posterior distribution of **${{\varvec{\varepsilon}}}_{{\varvec{p}}{\varvec{r}}{\varvec{i}}{\varvec{m}}{\varvec{a}}{\varvec{c}}{\varvec{y}}}$** and **${{\varvec{\varepsilon}}}_{{\varvec{r}}{\varvec{e}}{\varvec{c}}{\varvec{e}}{\varvec{n}}{\varvec{c}}{\varvec{y}}}$. (**A**) The mean of posterior distribution of ${\varepsilon }_{primacy}$ and ${\varepsilon }_{recency}$. Bars show the mean of subjects’ posterior means. Error bars represent standard errors across subjects. Each dot and line represent the mean of the posterior distribution of an individual subject. The difference between ${\varepsilon }_{primacy}$ and ${\varepsilon }_{recency}$ in the perceptual task (**B**) and the value-based task (**C**). Dots represents the mean and gray bars represent the 95% highest density interval. Subjects are sorted by the mean. (**D**) Temporal weighting function. The temporal weighting function $w(t)$ was computed using the posterior mean of ${\varepsilon }_{primacy}$ and ${\varepsilon }_{recency}$ of an individual subject. Then, $w(t)$ was averaged across subjects. The line shows the mean temporal weighting function. The shaded area represents standard errors across subjects. (**E**) Scatter plot of the difference between ${\varepsilon }_{primacy}$ and ${\varepsilon }_{recency}$ in the two tasks. Each dot represents the mean of an individual subject’s posterior distribution. The line represents the mean trend from linear regression

We observed a substantial recency bias in both the perceptual and value-based tasks. In the perceptual task, 34 of 38 subjects had higher values for ${\varepsilon }_{recency}$ than ${\varepsilon }_{primacy}$ (Figs. 5A-B). The 95% HDI of ${\varepsilon }_{primacy}-{\varepsilon }_{recency}$ was strictly negative and the posterior probability that ${\varepsilon }_{recency}$ was larger than ${\varepsilon }_{primacy}$ was 1 for these 34 subjects. By contrast, four subjects showed the opposite pattern, indicating a primacy bias. The 95% HDI of ${\varepsilon }_{primacy}-{\varepsilon }_{recency}$ was strictly positive and the posterior probability that ${\varepsilon }_{recency}$ was larger than ${\varepsilon }_{primacy}$ was 1 for these four subjects.

We observed similar results in the value-based task (Figs. 5A and C). As in the perceptual task, a majority of subjects—34 out of 38—had higher values for ${\varepsilon }_{recency}$ than ${\varepsilon }_{primacy}$. The 95% HDI of ${\varepsilon }_{primacy}-{\varepsilon }_{recency}$ was strictly negative and the posterior probability that ${\varepsilon }_{recency}$ was larger than ${\varepsilon }_{primacy}$ was 1 for these 34 subjects. By contrast, three subjects had a strictly positive HDI of ${\varepsilon }_{primacy}-{\varepsilon }_{recency}$ and a posterior probability that ${\varepsilon }_{primacy}$ was larger than ${\varepsilon }_{recency}$ equal to 1. For one subject, the 95% HDI included zero and the posterior probability that ${\varepsilon }_{recency}$ was larger than ${\varepsilon }_{primacy}$ was 0.66. While temporal bias for this subject cannot be definitively determined based on the 95% HDI, the posterior distribution supports a recency bias.

We also computed the temporal weighting function at the 30th stimulus pair (i.e., the last stimulus pair on each trial) using the posterior means of ${\varepsilon }_{primacy}$ and ${\varepsilon }_{recency}$. These temporal weighting functions clearly show that the weights on late stimulus pairs were higher than those on early stimulus pairs in both the perceptual and value-based tasks (Fig. 5D; see Fig. S1 for the subject-level functions).

The temporal weighting function was highly consistent across tasks. The means of ${\varepsilon }_{primacy}-{\varepsilon }_{recency}$ in the two tasks were positively correlated (Fig. 5E; r(36) = 0.66, *p* = 10^–5^). Consistent with the deviance results, this analysis indicates that subjects with a strong recency bias in one task also showed a strong recency bias in the other task.

**Fig. 6:**
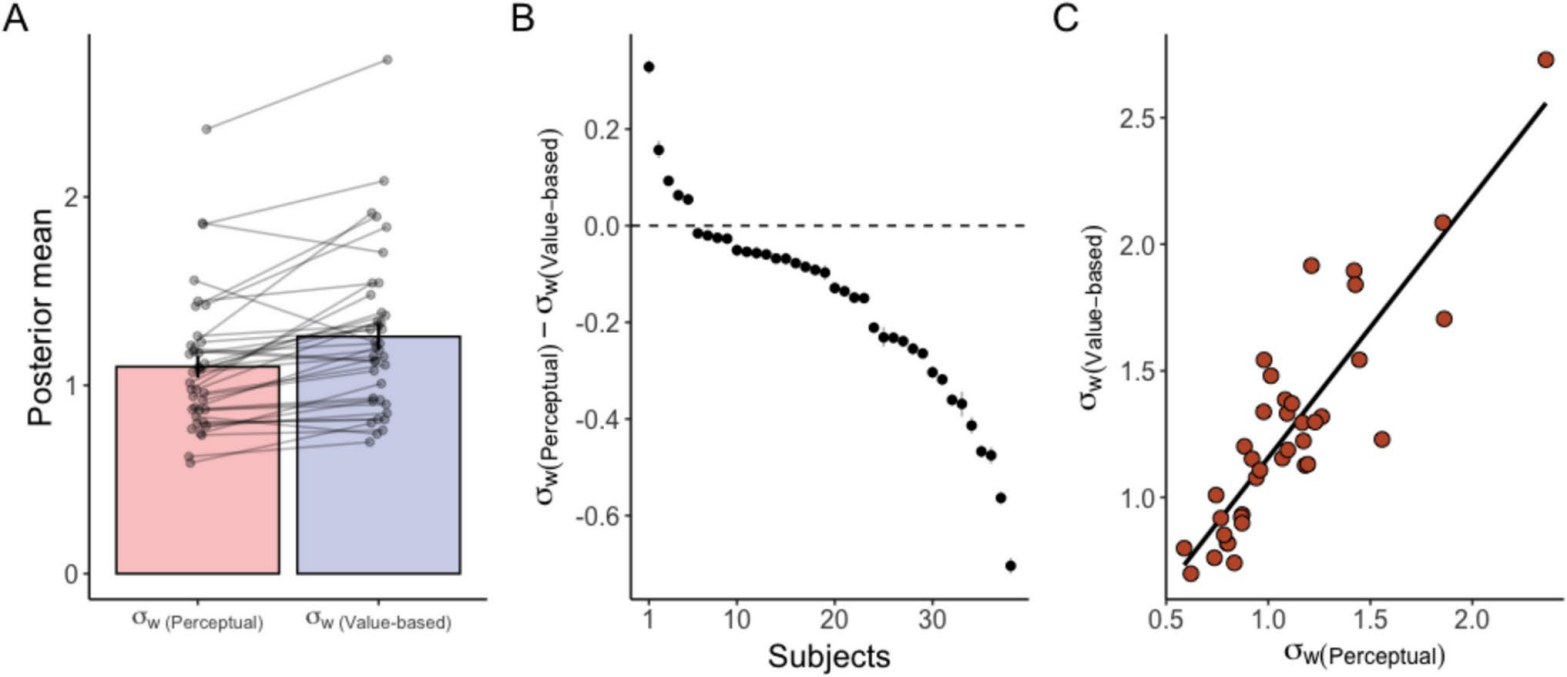
**Posterior distribution of **${{\varvec{\sigma}}}_{{\varvec{w}}}$**.** (**A**) The mean of the posterior distribution of ${\sigma }_{w}$. Bars show the mean of subjects' posterior means. Error bars represent standard errors across subjects. Each dot and line represent the mean of the posterior distribution of an individual subject. (**B**) The difference in ${\sigma }_{w}$ between the perceptual and the value-based tasks. The dots represents the means, and the gray bars represent the 95% highest density intervals. Subjects are sorted by the mean. (**C**) Scatter plot of ${\sigma }_{w}$ in the perceptual and the value-based tasks. Each dot represents the mean of an individual subject’s posterior distribution. The line represents the mean trend from  linear regression

In addition to the temporal bias, we examined the diffusion noise ${\sigma }_{w}$ in both decision domains. We assessed whether the 95% HDI of the difference in ${\sigma }_{w}$ between the perceptual and value-based tasks was positive or negative. Thirty-three subjects had strictly negative HDIs, indicating that they had larger ${\sigma }_{w}$ in the value-based task compared to the perceptual task (Figs. 6A-B). The posterior probability that ${\sigma }_{w}$ was larger in the value-based task than in the perceptual task was 1 for these 33 subjects. By contrast, five subjects had strictly positive HDIs, and a probability that ${\sigma }_{w}$ in the perceptual task was larger than in the value-based task equal to 1 (Figs. 6A-B). Also, the mean of the posterior distribution of ${\sigma }_{w}$ in the two tasks was positively correlated (Fig. 6C; r(36) = 0.88, *p* = 10^–13^). Thus, subjects with a smaller ${\sigma }_{w}$ in one task also tended to have a smaller ${\sigma }_{w}$ in the other task.

Lastly, we explored the relationship between subjects’ performance in the evidence-averaging task and the parameters of the ADM. Specifically, we examined the correlation between ME and the posterior means of ADM parameters. We found that ME in the evidence-averaging task was negatively correlated with the posterior mean of ${\varepsilon }_{primacy}-{\varepsilon }_{recency}$ in both tasks (perceptual task: r(36) =  − 0.34, *p* = 0.04; value-based task: r(36) =  − 0.37, *p* = 0.02). In other words, more error was correlated with more recency vs. primacy bias. Also, the ME and ${\sigma }_{w}$ were positively correlated in both tasks (perceptual task: r(36) = 0.66, *p* = 10^–6^; value-based task: r(36) = 0.88, *p* = 10^–13^).

## Neuroimaging results

### GLM1. The model with ADM-predicted AE

**Fig. 7:**
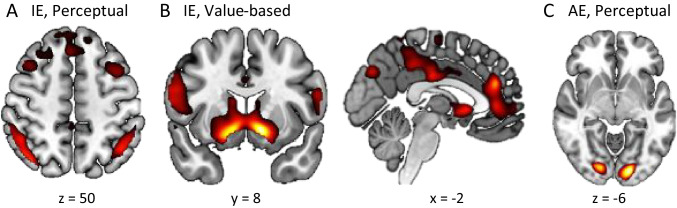
Brain regions tracking unsigned instantaneous evidence (IE) and average evidence (AE) (GLM1). Brain regions tracking the unsigned IE in the perceptual task (**A**) and the value-based task (**B**). (**C**) Brain regions tracking the unsigned ADM-predicted AE in the perceptual task. *P* values were set to .001, uncorrected for visualization. ADM: Averaging Diffusion Model

In the GLM with ADM-predicted AE, we found distinct brain regions that tracked the unsigned IE (Table S3) in the two tasks. In the perceptual task, the inferior parietal gyrus (x =  − 42, y =  − 58, z = 50, Z = 4.62; Fig. 7A) and angular gyrus (x = 42, y =  − 74, z = 40, Z = 6.30) tracked the unsigned IE. In the value-based task, both the ventral striatum (x =  − 12, y = 8, z =  − 8, Z = 5.86; Fig. 7B) and the medial prefrontal cortex (x =  − 2, y = 40, z = 24, Z = 4.96; Fig. 7B) tracked the unsigned IE.

Surprisingly, we only observed significant neural correlates with unsigned ADM-predicted AE in the perceptual task (Table S4). The occipital cortex (x =  − 16, y =  − 90, z =  − 6, Z = 5.12; x = 16, y =  − 86, z =  − 4, Z = 4.67; Fig. 7C) showed significant activation corresponding to the unsigned AE in the perceptual task. There was no significant cluster tracking the unsigned AE in the value-based task.

### GLM2. The model with behavioral AE

**Fig. 8:**
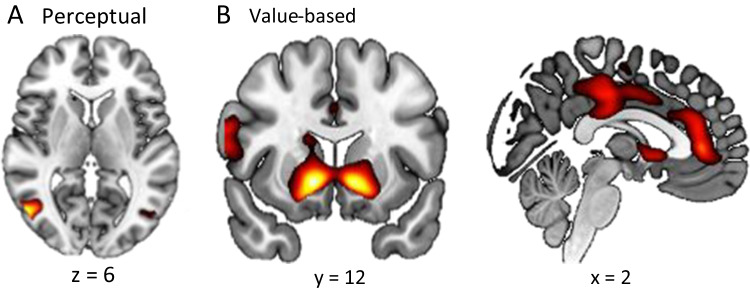
Brain regions tracking the unsigned instantaneous evidence (IE) (GLM2). Brain regions tracking the unsigned IE in the perceptual task (**A**) and the value-based task (**B**). *P* values were set to .001, uncorrected for visualization

As in GLM1, distinct brain regions were found to track the unsigned IE in the two tasks in GLM with behavioral AE (Table S5). In the perceptual task, the middle temporal gyrus (x = 52, y =  − 70, z = 6, Z = 4.86; Fig. 8A) and middle occipital cortex including angular gyrus (x = 40, y =  − 78, z = 36, Z = 4.52; Fig. 8A) tracked the unsigned IE. Like in GLM1, the ventral striatum (x = 12, y = 12, z =  − 6, Z = 5.61; Fig. 8B) and the ventromedial prefrontal cortex (x = 2, y = 40, z = 4, Z = 4.51; Fig. 8B) tracked the unsigned IE in the value-based task.

**Fig. 9:**
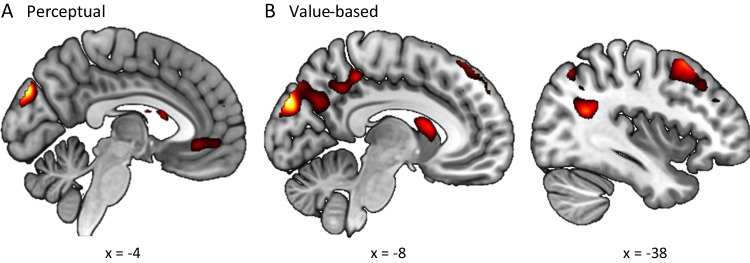
Brain regions tracking the unsigned behavioral average evidence (AE) (GLM2). Brain regions tracking the unsigned behavioral AE in the perceptual task (**A**) and the value-based task (**B**). *P* values were set to .001, uncorrected for visualization

Common and distinct brain regions tracked the unsigned behavioral AE in the two tasks (Table S6). The cuneus tracked the unsigned behavioral AE in both the perceptual task (x =  − 4, y =  − 84, z = 34, Z = 4.82; Fig. 9A) and the value-based task (x =  − 8, y =  − 90, z = 32, Z = 5.23; Fig. 9B). Additionally, the left dorsolateral prefrontal cortex (x =  − 38, y = 16, z = 56, Z = 4.43; Fig. 9B) tracked the unsigned behavioral AE in only the value-based task.

### ROI analysis

**Fig. 10:**
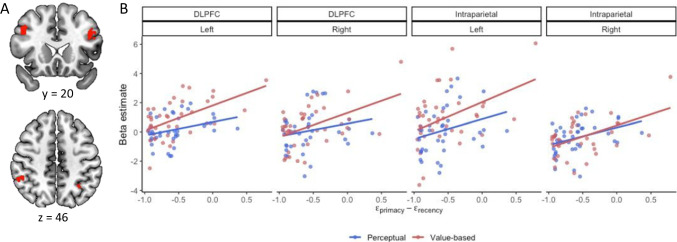
Region of interest (ROI) analysis results. (**A**) ROIs created from a term-based meta-analysis at Neurosynth. Bilateral DLPFC (top) and intraparietal sulcus (bottom) were chosen as our ROIs. (**B**) The correlation between beta estimates for the stimulus onset regressor in GLM1 and the difference between primacy and recency parameters. Dots represent individual subjects’ data and lines represent mean trends from linear regression

We found that the brain regions associated with cognitive control reflected individual differences in temporal bias. Bilateral DLPFC and left intraparietal sulcus were more active at stimulus onset for individuals with a stronger primacy bias in the value-based task (left DLPFC: r(36) = 0.55, *p* < 0.001; right DLPFC: r(36) = 0.47, *p* = 0.003; left intraparietal sulcus: r(36) = 0.43, *p* = 0.008; Fig. 10). In the right intraparietal sulcus, activity at stimulus onset was higher for individuals with a stronger primacy bias in both perceptual and value-based tasks (perceptual r(36) = 0.35, *p* = 0.03; value-based r(36) = 0.05, *p* = 0.001; Fig. 10). This result suggests that individuals with stronger primacy biases recruit more cognitive control when averaging evidence, perhaps to inhibit evidence from later in the stimulus sequence.

In the supporting information, we also report the results of a psychophysiological interaction (PPI) analysis to identify brain regions whose activity depends on the activity of brain regions tracking the unsigned IE.

## Discussion

We investigated temporal bias in the evidence-averaging process. In particular, we aimed to understand how temporal bias affects the continuous updating of average evidence over time. We found that people generally exhibit substantial recency bias but only a little primacy bias in their estimates of average evidence. We established these biases in two domains: perceptual and value-based choice. These biases are highly consistent within an individual; people who are more recency-biased in one domain are also more recency-biased in the other. In the value-based, but not perceptual task, we found that the dlPFC tracks the average evidence. Finally, we found that a brain network associated with cognitive control (dlPFC and intraparietal sulcus) is more active for people who exhibit relatively more primacy bias than recency bias.

Our experiment directly examines temporal biases in information processing, rather than indirectly inferring it from choices (Cheadle et al., 2014; Tsetsos et al., 2011, 2012; Wyart et al., 2012). By having subjects continuously update their estimates on a continuous scale, we could infer their temporal weighting functions from their continuous reports. Our results qualitatively replicate past work, showing the prevalence of recency bias and a small proportion of primacy bias. Our approach allows for a more granular evaluation of the relative impacts of primacy and recency biases in the evidence-averaging process.

With this approach, we identified strong consistency of the temporal weighting function within individuals, across different domains. While the best-fitting model supported different parameters in the two tasks, subjects who showed a strong recency bias in the perceptual task also showed a strong recency bias in the value-based task. The stability of the temporal weighting function indicates that temporal bias is not confined to a specific decision domain. This lends further credence to the idea that perceptual and value-based decisions are governed by overlapping principles (Frydman & Nave, 2017; Glimcher, 2022; Polanía et al., 2014; Shadlen & Shohamy, 2016; Summerfield & Tsetsos, 2012; Smith & Krajbich, 2021).

Although temporal bias is relatively stable within an individual, it does vary substantially across individuals. The majority of subjects showed a strong recency bias, putting more weight on recent evidence in their evaluations. Conversely, a smaller number of subjects showed a strong primacy bias, putting more weight on early evidence in their evaluations. The prevalence of recency bias in our task may reflect a misapplied adaptive decision-making strategy that only works for prospective judgments. The stimulus sequence in our study was not generated by sampling from a single distribution; it was generated from a random walk. Thus, our stimulus sequence mimics a volatile environment. In such an environment, assigning greater weights to the most recent decision evidence is an adaptive decision strategy when making prospective judgments (Behrens et al., 2007; Hubert-Wallander & Boynton, 2015). It is likely that people are more familiar with making prospective judgments (i.e., predicting the future) and incorrectly apply strategies from that setting to retrospective judgments.

Another reason for recency bias in retrospective judgment may be limited memory capacity. To compute average evidence (AE), a decision maker may try to store individual evidence samples and compute their average. When memory capacity is limited, early evidence might be discarded to store and process the latest evidence. On the one hand, limited memory could be a major issue in our study as we presented long sequences of stimuli. Conversely, subjects in our study could also have avoided the memory burden by using the on-screen slider bar to store prior evidence. The fact that we observed neural correlates of AE, at least in the value-based task, suggests that subjects did mentally track AE and thus could have faced memory constraints, which could have contributed to the pronounced recency bias in our study.

Although primacy opposes recency, they are not mutually exclusive, and a primacy bias may also have its advantages. As a trial progresses, each new sample has a smaller impact on the average. Thus, a decision maker might choose to save cognitive resources by focusing on the stimuli early in a trial and then later inhibiting further updates. Our fMRI results support this explanation. We found that activity in brain regions associated with cognitive control correlate with individual differences in temporal bias. Specifically, bilateral DLPFC and intraparietal sulcus were more active for individuals with a stronger primacy vs. recency bias. This suggests that the primacy biases that we observed were a deliberate strategy on the part of our subjects. This behavior might be analogous to people who fail to update their beliefs once they have formed a strong initial impression (Fourakis & Cone, 2020; Sullivan, 2019).

Primacy bias is also a feature of some sequential sampling models, such as the Ornstein–Uhlenbeck model and the leaky competing accumulator model (Polanía et al., 2014; Usher & McClelland, 2001). There is considerable support for these models (Busemeyer & Townsend, 1993; Roe et al., 2001; Tsetsos et al., 2011; Turner et al., 2018), consistent with the results from our study.

Earlier studies have accounted for temporally weighted evidence-averaging processes with population-coding and evidence-accumulation frameworks (Brezis et al., 2015; Bronfman et al., 2016; Keung et al., 2020). Brezis & colleagues (2015) propose a dual-component model of numerical averaging in which the number of items to be averaged influences the computational mechanism. They assumed that a greater number of items would lead cognitive agents to use more “intuitive” neurophysiological evidence provided by the population code than an analytic solution. A dynamic leaky competing accumulator model (Bronfman et al., 2016) accounts for the primacy and recency effects as emergent properties of its evidence accumulation mechanism, assuming increasing leakage and decreasing inhibition over time. Leaky inhibitory mechanisms can also implement divisive normalization (Keung et al., 2020).

Both approaches account for the temporal bias as a product of decision processes. For example, population-coding approaches (Brezis et al., 2015, 2016, 2018) can use the decay of neural activations induced by earlier stimuli to realize the recency effect. The Bronfman model does not calculate the arithmetic mean of evidence; instead, the leak and inhibition in the evidence accumulation dynamics determine the relative contribution of evidence from individual stimuli. The Keung model further provides a method to quantify the temporal weighting function directly from the model.

Compared with the Bronfman model, we use a mathematical function that directly calculates the item-wise temporal weight (Pooley et al., 2011). This function does not provide a mechanistic account of temporal weighting (unlike the Bronfman model) but allows us to evaluate and incorporate the item-wise weights seamlessly within the ADM. Some of the aforementioned studies (Brezis et al., 2015; Bronfman et al., 2016) report temporal weighting profiles, but they are calculated from behavioral data in a post-hoc manner.

The temporal weighting scheme in our model (Turner et al., 2017) relies on a process of value averaging that is similar to the mechanism of divisive normalization. Divisive normalization has been proposed as a way to reweight neural activations over time and has been observed in several brain areas (Carandini & Heeger, 2012; Pouget et al., 2013). Many authors have argued that divisive normalization could be performed through the neural computations of inhibition and leakage (Keung et al., 2020; Louie et al., 2014).

Our study also revealed distinct neural mechanisms involved in evaluating the current evidence and tracking the average evidence over time. We found minimal representation of the average evidence signals; aside from the dlPFC in the value-based task, the main result was activity in visual cortex, presumably having to do with the position of the slider bar. However, we found sensible, domain-specific representations of the current evidence in posterior parietal cortex for the perceptual task and in vmPFC and ventral striatum for the value-based task. Parietal lobe is involved in processing numbers (Leibovich et al., 2017); it shows higher activation when comparing Arabic numbers or the quantities of visual stimuli (Cantlon et al., 2009; Harvey et al., 2013; Kanayet et al., 2014). Thus, the involvement of the parietal lobe is reasonable given that subjects were judging the number of white squares in the display. The vmPFC and ventral striatum are part of the reward network (Bartra et al., 2013; Clithero & Rangel, 2014) and represent subjective value in many decisions, including those based on sequential sampling (Gluth et al., 2012; Hare et al., 2011; Pisauro et al., 2017; Rodriguez et al., 2015). Consistent with these findings, subjects in our study recruited the reward system to evaluate the relative value of the snack foods.

One reason that we may have struggled to find neural representations of AE is the high correlation between IE and AE in the observed responses. We designed the experiment to minimize the correlation between IE and AE, assuming no temporal bias. However, the prevalence of recency bias resulted in a strong correlation between IE and AE in the majority of subjects. The high collinearity between these two regressors may have made it difficult to disentangle the neural signals associated with each process. To address this limitation in future studies, a potential approach would be to adaptively generate sequences for each subject based on their estimated temporal bias, thereby minimizing the correlation between IE and AE (Cavagnaro et al., 2010; Myung & Pitt, 2009).

It is also worth noting that the fMRI results from our model-based GLM1 did not reveal as much as our behavior-based GLM2. As noted above, one reason for this might be the high correlation between IE and AE for many subjects. The noise in behavior might have helped to decorrelate these two measures. An alternative explanation is that our model does not adequately capture subjects’ behavior. Comparisons between behavior and our simulated model indicate a generally good fit, but there was of course heterogeneity in how well the model captured each subject’s behavior. A poor fit for even a few subjects could be pivotal in an fMRI with a limited sample size.

In our task, participants were asked to explicitly track and report the average evidence over the course of each trial. It is possible that the cognitive and neural mechanisms would have been different if we had only asked subjects to report the average evidence at the end of each trial. Thus, the scope of our findings is limited to cases where people are continuously tracking the average over time, as in our motivating example of the college football rankings.

Retrospective evaluation is an important aspect of our lives. We often look back in time to pinpoint the relationships among events in the past and events in the present. Often, this requires a careful and equal consideration of each event in the past. Our results indicate that people are typically bad at doing this. Most people tend to put too much weight on recent information, and another smaller group tend to put too much weight on the earliest information (i.e., jumping to conclusions). Both strategies lead to substantial distortions of the truth, distortions that appear to be stable across both perceptual and value-based domains.

## Supplementary Information

Below is the link to the electronic supplementary material.

Supplementary file1 (PDF 484 KB)

## Data Availability

All behavioral data is available on OSF (https://osf.io/38ugk/).

## Code availability

All code for analysis is available on OSF (https://osf.io/38ugk/).
