# Supplementary material for: People display consistent recency and primacy effects in behavior and neural activity across perceptual and value-based judgments
Source: Cogn Affect Behav Neurosci. 2025 Mar 26;25(4):923–40. doi: 10.3758/s13415-025-01285-1 (PMC12356758; doi:10.3758/s13415-025-01285-1)
Supplement: Supplementary file 1 — Supplementary file1 (PDF 484 KB) [file 13415_2025_1285_MOESM1_ESM.pdf]

People display consistent recency and primacy effects in behavior and neural activity across perceptual and value-based judgments

Supporting Information

Minhee Yoo[1], Giwon Bahg[2], Brandon Turner[1], Ian Krajbich[1, 3, 4,+]

[1]Department of Psychology, The Ohio State University, Columbus, OH, United States of America

[2]Department of Psychology, Vanderbilt University, Nashville, TN, United States of America

[3]Department of Economics, The Ohio State University, Columbus, OH, United States of America

[4]Department of Psychology, University of California Los Angeles, Los Angeles, CA, United States of America

[+]Corresponding author

E-mail: krajbich@ucla.edu

Supplementary Results

Psychophysiological interaction analysis

We conducted a psychophysiological interaction (PPI) analysis to identify brain regions whose activity depends on the activity of brain regions tracking the unsigned IE. We chose brain regions tracking the unsigned IE found using GLM1 (the model with ADM-predicted AE) as ROIs. Specifically, we extracted brain responses from angular gyrus (x = 42, y = -74, z = 40) in the perceptual task and ventral striatum (x = -12, y = 8, z = -8) in the value-based task. We constructed a separate GLM for each task with three regressors: the onset of stimulus, brain response from a ROI, and the interaction between brain response and stimulus onset. We also included six realignment parameters that were generated during preprocessing.

We created positive contrast images for the interaction regressor at the subject-level and then tested them with one sample t-tests at the group-level. We defined clusters at a p-value of .001 and further corrected the p-values based on the size of clusters or the peak activation of a cluster.

In the perceptual task, we found that activity in the superior parietal lobe (x = -16, y = -60, z = 58; Z = 4.55) and occipital lobe (x = -26, y = -86, z = 14; Z = 4.5) was modulated by the activity of angular gyrus. In the value-based task, activity in the occipital lobe (x = 34, y = -88, z = 16, Z = 4.83; x = -24, y = -82, z = -10, Z = 4.57) varied depending on the activity of ventral striatum. In both tasks, these brain regions showed a higher activation when the activity of the respective ROI was higher.

Supplementary Figure 1. Temporal weighting function of individual subjects.

This figure shows the temporal weighting function at the 30th stimulus pair (i.e., the last stimulus pair on each trial). Each panel shows an individual subject's temporal weighting function in the perceptual (blue) and value-based task (red). The temporal weighting function $w(t)$ was computed using the posterior mean of $\varepsilon_{primacy}$ and $\varepsilon_{recency}$ of an individual subject.

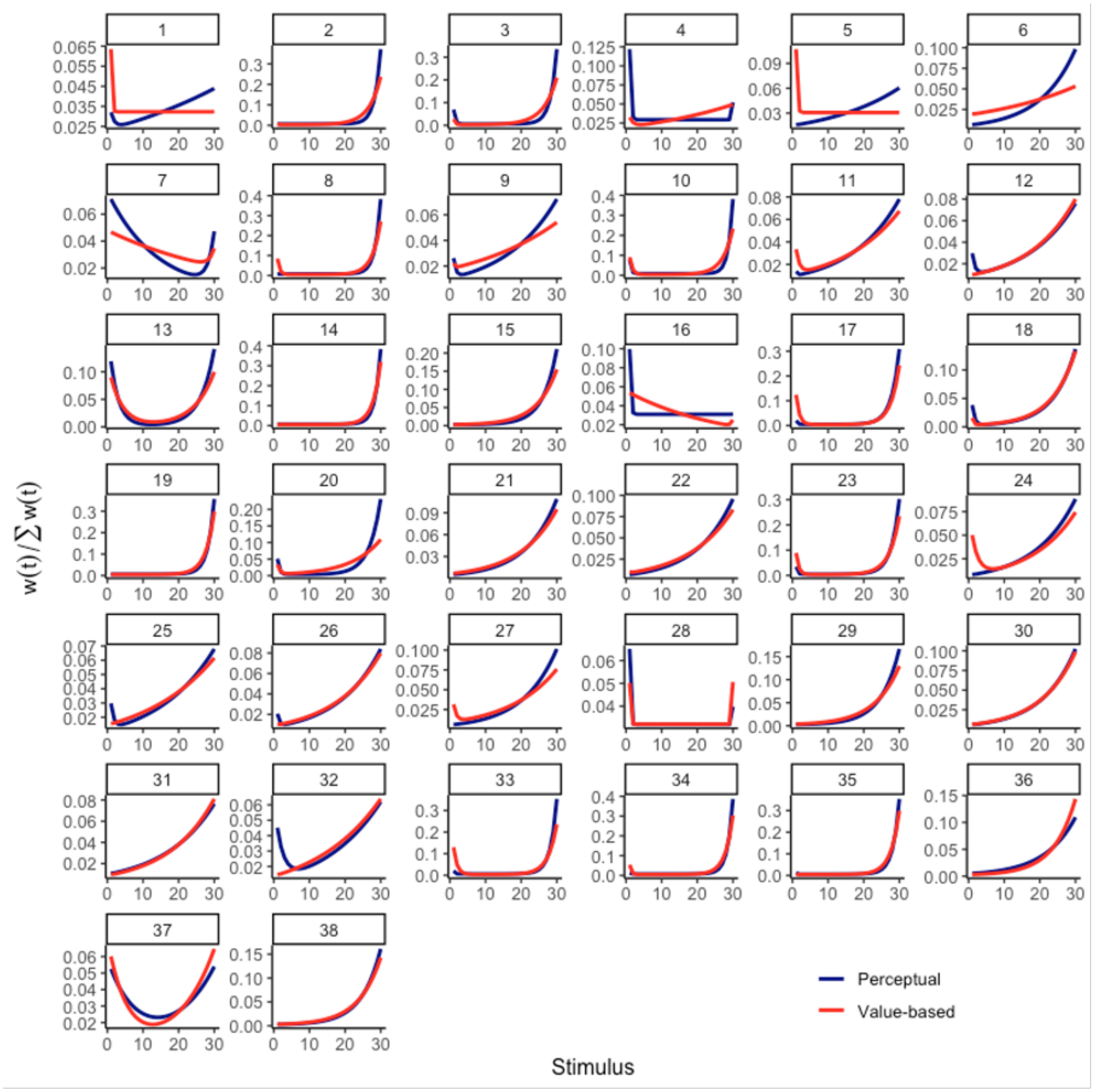

Supplementary Table 1. Distribution of widely applicable information criterion (WAIC) across subjects.

This table summarizes the mean, median, and first and third quantiles of the widely applicable information criterion (WAIC) of four variants of the Averaging Diffusion Model. A lower WAIC indicates a better fit of a model to the data.

	Temporal bias	Noise	Mean	Median	Q1	Q3
Model 1	Separate	Separate	15885.33	19287.00	-18101.05	32351.44
Model 2	Common	Separate	17396.54	20921.42	-16682.24	33706.56
Model 3	Separate	Common	17325.17	20553.18	-17707.66	35643.11
Model 4	Common	Common	18890.55	20807.26	-16618.19	36973.16

Supplementary Table 2. Distribution of mean of posterior samples across subjects.

This table summarizes the mean of posterior samples from the separate-temporal-bias and separate-noise model across subjects. It shows the mean, median, first and third quartiles, and standard deviation of the posterior sample mean.

	Perceptual task			Value-based task		
	$\varepsilon_{primacy}$	$\varepsilon_{recency}$	σ_w	$\varepsilon_{primacy}$	$\varepsilon_{recency}$	σ_w
Mean	0.14	0.71	1.10	0.19	0.74	1.26
Median	0.02	0.83	1.04	0.02	0.85	1.19
Q1	0.00	0.56	0.87	0.00	0.66	0.92
Q3	0.15	0.92	1.21	0.29	0.93	1.38
SD	0.24	0.27	0.36	0.28	0.27	0.43

Supplementary Table 3. Brain regions tracking the unsigned instantaneous evidence (GLM1).

We defined clusters at a p-value of .001 and further corrected the p-values based on the size of clusters or the peak activation of a cluster at p-value <.05. Each coordinate was labeled with the AAL3 (Anatomical Automatic Labeling 3) toolbox in SPM12.

**Perceptual task**								
**Label**	**Side**	**x**	**y**	**z**	**Z**	**cluster size**	**cluster p-value**	**peak p-value**
Angular gyrus	R	42	-74	40	6.295	20336	<.001	<.001
Precuneus	R	22	-46	14	5.138	409	.052	.003
Crus II of cerebellar hemisphere	R	20	-88	-36	5.015	522	.025	.005
Inferior parietal gyrus	L	-42	-58	50	4.623	3979	<.001	.027
Crus II of cerebellar hemisphere	L	-10	-88	-36	4.252	430	.045	.108
**Value-based task**								
**Label**	**Side**	**x**	**y**	**z**	**Z**	**cluster size**	**cluster p-value**	**peak p-value**
Putamen	L	-12	8	-8	5.858	8298	<.001	<.001
Insula	L	-44	-2	6	5.525	3390	<.001	<.001
Anterior cingulate cortex, pregenual	L	-2	40	24	4.956	1841	<.001	.006
Middle cingulate & paracingulate gyri	R	6	-28	38	4.49	2343	<.001	.037
Inferior frontal gyrus, triangular part	R	52	40	-2	4.075	502	.045	.160

Supplementary Table 4. Brain regions tracking the unsigned average evidence (GLM1).

We defined clusters at a p-value of .001 and further corrected the p-values based on the size of clusters or the peak activation of a cluster at p-value <.05. Each coordinate was labeled with the AAL3 (Anatomical Automatic Labeling 3) toolbox in SPM12.

**Perceptual task**								
**Label**	**Side**	**x**	**y**	**z**	**Z**	**cluster size**	**cluster p-value**	**peak p-value**
Middle occipital gyrus	L	-16	-90	-6	5.122	835	.008	.003
Lingual gyrus	R	16	-86	-4	4.671	529	.036	.019

Supplementary Table 5. Brain regions tracking the unsigned instantaneous evidence (GLM2).

We defined clusters at a p-value of .001 and further corrected the p-values based on the size of clusters or the peak activation of a cluster at p-value <.05. Each coordinate was labeled with the AAL3 (Anatomical Automatic Labeling 3) toolbox in SPM12.

**Perceptual task**								
**Label**	**Side**	**x**	**y**	**z**	**Z**	**cluster size**	**cluster p-value**	**Peak p-value**
Middle temporal gyrus	R	52	-70	6	4.86	372	.044	.013
Middle occipital gyrus	R	40	-78	36	4.519	430	.028	.050
**Value-based task**								
**Label**	**Side**	**x**	**y**	**z**	**Z**	**cluster size**	**cluster p-value**	**peak p-value**
Ventral Striatum	R	12	12	-6	5.61	2128	<.001	<.001
Supramarginal gyrus	R	50	-34	42	4.793	1535	<.001	.015
Rolandic operculum	L	-44	-2	8	4.662	732	.005	.026
Anterior cingulate cortex, pregenual	R	2	40	4	4.513	1467	<.001	.047
Inferior frontal gyrus, opercular part	R	58	8	2	4.365	599	.011	.082
Middle cingulate & paracingulate gyri	L	-2	-22	40	4.33	1965	<.001	.093
Middle temporal gyrus	R	46	-66	4	4.266	432	.034	.115
Inferior parietal gyrus	L	-58	-22	48	4.187	1110	.001	.151

Supplementary Table 6. Brain regions tracking the unsigned average evidence (GLM2).

We defined clusters at a p-value of .001 and further corrected the p-values based on the size of clusters or the peak activation of a cluster at p-value <.05. Each coordinate was labeled with the AAL3 (Anatomical Automatic Labeling 3) toolbox in SPM12.

**Perceptual task**								
**Label**	**Side**	**x**	**y**	**z**	**Z**	**cluster size**	**cluster p-value**	**peak p-value**
Cuneus	L	-4	-84	34	4.82	307	.113	.012
**Value-based task**								
**Label**	**Side**	**x**	**y**	**z**	**Z**	**cluster size**	**cluster p-value**	**peak p-value**
Cuneus	L	-8	-90	32	5.234	3102	<.001	.002
Middle frontal gyrus	L	-38	16	56	4.425	890	.001	.071
Middle temporal gyrus	L	-42	-42	0	3.851	516	.015	.424

Supplementary Table 7. Correlation between the activity in the four ROIs and the posterior means of parameters.

The table shows the correlation between the activity in the four regions of interest and the posterior means of $\varepsilon_{primacy}$ and $\varepsilon_{recency}$ parameters.

		**Perceptual task**				**Value-based task**			
**ROI**	**Side**	$\varepsilon_{primacy}$		$\varepsilon_{recency}$		$\varepsilon_{primacy}$		$\varepsilon_{recency}$	
		**r**	**p**	**r**	**p**	**r**	**p**	**r**	**p**
**DLPFC**	**L**	0.2	.22	-0.19	.25	0.41	.01	-0.41	.01
	**R**	0.07	.66	-0.18	.27	0.27	.12	-0.44	.006
**Intraparietal sulcus**	**L**	0.02	.89	-0.32	.05	0.3	.06	-0.33	.04
	**R**	0.1	.57	-0.34	.04	0.39	.02	-0.36	.03

Supplementary Table 8. Deviation of subjects' responses from the simple average evidence as a function of time.

The table shows the results of a linear regression on the error in the evidence-averaging task. Time within a trial, task type (value-based vs. perceptual), and their interaction were included as regressors. Standard errors were clustered at the subject level.

	B	**SE**	***t***	**p**
Intercept	0.22	0.01	25.59	<.001
Time in a trial	0.003	0.001	3.53	<.001
Task type (value-based)	0.02	0.01	3.24	.001
Time in a trial * Task type (value-based)	-0.002	0.0005	-3.85	<.001

Supplementary Table 9. The influence of instantaneous evidence on average evidence updates.

The table shows the linear regression results on average evidence (AE) updates. AE updates were defined as the difference between the last slider positions of two consecutive stimulus pairs. Instantaneous evidence (IE), time within a trial, task type (value-based vs. perceptual), and all their interactions were included as regressors. Standard errors were clustered at the subject level.

	B	**SE**	***t***	**p**
Intercept	-0.01	0.001	-4.73	<.001
IE	0.18	0.01	17.34	<.001
Time in a trial	0.0004	0.0001	5.18	<.001
Task type (value-based)	0.001	0.001	1.32	.19
IE * Time in a trial	-0.005	0.0003	-14.70	<.001
IE<br>* Task type (value-based)	-0.04	0.01	-6.57	<.001
Time in a trial<br>* Task type (value-based)	-0.00008	0.0001	-1.12	.26
IE * Time in a trial<br>* Task type (value-based)	0.001	0.0002	6.71	<.001
